# The frequency of MEFV gene variations in Adult-onset Still's disease and Gout

**DOI:** 10.1186/1546-0096-13-S1-P15

**Published:** 2015-09-28

**Authors:** S Ugurlu, AS Emekli, E Tahir Turanli, SG Benyakar, G Çelikyapı Erdem, H Ozdogan, E Seyahi

**Affiliations:** 1 Cerrahpasa Medical Faculty, University of Istanbul, Division of Rheumatology, Department of Internal Medicine, Istanbul, Turkey; 2 Science and Letters Faculty, Istanbul Technical University, Molecular Biology and Genetics Department, Istanbul, Turkey

## Objectives

Adult onset-Still's disease (AOSD) and gout are considered as auto-inflammatory disorders. Both diseases run recurrent episodic course and respond to anti-IL 1 treatment. Additionally, increased frequency of MEFV variations in other inflammatory diseases other than FMF such as Behçet's disease, ulcerative colitis and rheumatoid arthritis and raises the possibility that MEFV gene may play a general role in the inflammatory pathway. Therefore in this study, we explored the MEFV exon 2 and 10 variations in a group of AOSD and gout patients and compared the frequencies between disease groups and healthy controls.

## Patients and methods

We studied all consecutive 42 patients with FMF (mean age: 33.3 ± 15.2), 28 patients with adult onset Still's disease (mean age: 37.9±8.4), 29 patients with gout (mean age: 50.3 ± 9.7), and 44 healthy controls (mean age: 33.6±10.2).

Genomic DNA was isolated from venous blood, using basic salting-out technique. PCR amplifications were done in three sets of primers covering exon 2 and exon 10 regions. Gel purified products were Sanger sequenced and chromatograms were analysed using Genious Software by two independent researchers. MEFV variation frequencies were calculated using chi-square analysis.

## Results

The frequency of common exon 2 variation E148Q was found to be similar between the study groups (FMF: 5%, AOSD: 4%, gout: 3% and healthy controls: 3%). In exon 2, only R202Q variation was significantly more frequent in FMF group (43%) compared to other groups (18-25%) (*P=0.004*).

There was also significant difference in pathogenic exon 10 variations between FMF and other groups. The most prominent of these variations, M694V, was significantly more common in FMF group (49%), compared to AOSD (2%), gout (7 %) and healthy controls (1 %) (P<0.0001). The frequency of non-synonymous variations such as D102D-G138G-A165A, the common haplotype, was more likely to be more common in FMF group (66%) compared to AOSD (22%), gout (30%) and healthy controls (38%) (p< 0.05).

## Conclusions

AOSD and gout do not seem to be associated with MEFV gene mutations.

